# Giardiasis in urban and rural Amazonas, Brazil is driven by zoonotic and cosmopolitan A and B assemblages

**DOI:** 10.1590/0074-02760210280

**Published:** 2022-02-18

**Authors:** Lisiane Lappe dos Reis, Túllio Romão Ribeiro da Silva, Francisco Carlos de Oliveira Braga, Naara Macedo do Nascimento, Katia Maria Lima de Menezes, Alessandra Ferreira Dales Nava, Natália Aparecida de Souza Lima, Ana Carolina Paulo Vicente

**Affiliations:** 1 Fundação Oswaldo Cruz-Fiocruz, Instituto Leônidas & Maria Deane, Laboratório de Diversidade Microbiana da Amazônia de Importância para a Saúde, Manaus, AM, Brasil; 2 Fundação Oswaldo Cruz-Fiocruz, Instituto Leônidas & Maria Deane, Laboratório de Ecologia de Doenças Transmissíveis na Amazônia, Manaus, AM, Brasil; 3 Fundação Oswaldo Cruz-Fiocruz, Instituto Leônidas & Maria Deane, Manaus, AM; 4 Superintendência do Ibama no Amazonas, Centro de Triagem de Animais Silvestres, Manaus, AM, Brasil; 5 Fundação Oswaldo Cruz-Fiocruz, Instituto Oswaldo Cruz, Laboratório de Genética Molecular de Microrganismos, Rio de Janeiro, RJ, Brasil

**Keywords:** Giardia duodenalis, genotype, animals, human, TPI, BG

## Abstract

**BACKGROUND:**

*Giardia duodenalis* is a protozoan parasite that infects humans and other mammals and causes giardiasis worldwide. *Giardia* is genotyped into eight assemblages (A-H), with assemblages A and B considered zoonotic.

**OBJECTIVES:**

The aim of this study was to determine the assemblages of *G. duodenalis* from individuals living in rural and urban areas of the Amazonas State.

**METHODS:**

103 human faecal specimens microscopically positive for the presence of *Giardia* obtained from four municipalities in Amazonas and four animal faecal specimens were genotyped based on the sequences of two genes, triosephosphate isomerase (*TPI*) and β-giardin (*BG*).

**FINDINGS:**

In humans, assemblage A was the most represented with the identification of sub-assemblages AI, AII and AIII based on *BG* and sub-assemblages AI and AII based on *TPI*. Similarly, there is a diversity of sub-assemblage B considering *BG* (B and BIII) and *TPI* (B, BIII and BIV). In addition, we characterised homogeneous and heterogeneous genotypes comprising assemblages/sub-assemblages A and B in individuals from urban and rural areas. Here, for the first time, it was genotyped *Giardia* that infects animals from the Brazilian Amazon region. We identified sub-assemblage AI in one *Ateles paniscus* and two *Felis catus* and sub-assemblage BIV in one *Lagothrix cana*.

**MAIN CONCLUSIONS:**

Therefore, humans and animals from the urban and rural Amazon share *Giardia* genotypes belonging to assemblages A and B, which are found in cosmopolitan regions around the world.

*Giardia duodenalis* (syn. *G. intestinalis* or *G. lamblia*) is a protozoan parasite that infects the upper intestinal tract of humans and other animals, causing giardiasis worldwide, which is considered a neglected disease by World Health Organization (WHO).^1^ Symptoms in humans such as acute diarrhea may progress to a chronic stage, but most infections remain asymptomatic.^2^ In children, giardiasis has a negative impact on their growth and cognitive development.^3^ *G. duodenalis* is phylogenetically classified into eight assemblages (A to H). Assemblages A and B are zoonotic, and in humans, the prevalence of assemblage B is higher in both low/high-income areas and in different age groups in the world, the exception is Australia where assemblage A is the prevalent.^4^ In other mammals, assemblages C and D are specific to dogs and other canids, assemblage E is found in ungulates including livestock, assemblage F is found in cats, assemblage G is found in rodents, and assemblage H is found in marine mammals, such as pinnipeds family.^5^ However, this is a dynamic scenario as recently assemblages C, E and F have also been characterised in human infections.^4^ ^,^ ^6^ ^,^ ^7^ Based on single nucleotide polymorphisms, assemblages A and B have been further defined into sub-assemblages and subtypes,^5^ revealing great diversity in *G. duodenalis*. In Brazil, a continental country characterised by many biomes, assemblages A and B have been described with different prevalence in humans and animals, although studies have been limited to a few human groups, mainly Southeast Brazil.^8^

As for the Amazon biome, the prevalence of assemblage B has been noted in two studies conducted in this region based on small indigenous groups.^9^ ^,^ ^10^ To date, there is a gap in terms of animal studies on the occurrence of *Giardia* in this biome.

Here, we performed a genetic characterisation of *G. duodenalis* in individuals living in rural and urban areas in the state of Amazonas, and found a prevalence of assemblage A over assemblage B. Our study also included animals, and as in humans, assemblage A predominated.

## MATERIALS AND METHODS

**Fig. 1:**
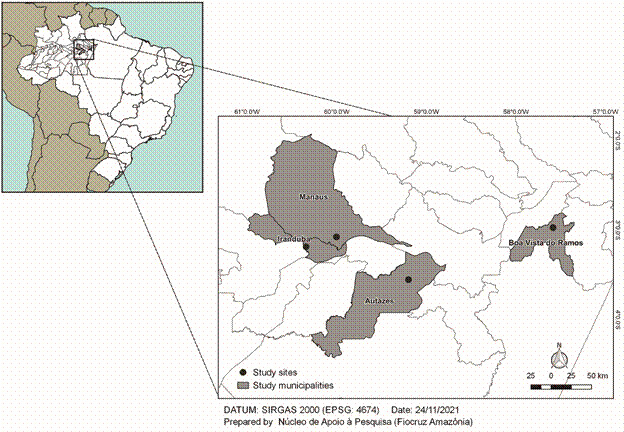
location of the four study sites, municipalities of Manaus, Boa Vista do Ramos, Iranduba (Lago do Limão community) and Autazes (São Félix community), in Amazonas, Brazil.

*Study area* - The study was performed based on samples from four municipalities located in Amazonas State, Brazil, which belong to the Amazon biome (Fig. 1). One urban area: (Manaus (n = 53) - (3º4’25”S, 60º0’20”W), and the three others rural areas: Iranduba (n = 24) - (Lago do Limão rural community; 03º11’0.99”S, 60º20’35.89”W), Autazes (n = 15) - (São Félix rural community; 03º33’02.8”S, 059º12’05.8”W) and Boa Vista do Ramos (n = 11) - (2º58’12”S, 57º35'24"W), from 2018 to 2019.

*Obtaining and analysing stool samples* - We obtained, for convenience, 103 human stool sediments positive for *G. duodenalis* of the public health laboratories from these four municipalities and the presence of *Giardia* was confirmed by microscope observation. Besides, we performed parasitological examination by spontaneous sedimentation and centrifugal-flotation in zinc sulfate^11^ ^,^ ^12^ in 14 animal faecal specimens: (n = 4) are from *Ateles paniscus* - black spider monkey and *Lagothrix cana* - gray wooly monkey (n = 2) non-human-primates (NHP) from Wild Animal Rehabilitation Centre (CETAS)/IBAMA and companion cats (n = 8) from a veterinary clinic in urban area/Manaus. Cats had no gastrointestinal symptoms. The cats’ guardians did not want to perform the parasitological analysis of their stool.

*Dna extraction, polymerase chain reaction (PCR) and sequencing* - DNA was extracted from *Giardia* positive samples using QIAamp DNA Stool Mini Kit with minor modifications: lysis buffer temperature to 95ºC for 15 min, and 200 uL of elution buffer for 10 min at room temperature. PCR was carried out targeting the triosephosphate isomerase (*TPI*)^13^ and β-giardin (*BG*)^14^ ^,^ ^15^ genes. The amplicons were purified using PureLink Quick PCR Purification Kit (Invitrogen, Lithuania), according to the manufacturer’s instructions. The fragments were Sanger sequenced using BigDye Terminator Cycle Sequencing Ready Reaction Kit.

*Data analysis* - The nucleotide sequences were edited in BioEdit software and the consensus sequences were aligned in ClustalW and the phylogenetic analyses were carried out in MEGAX software. The phylogenetic analyses were performed with sequences from humans and animals belonging to *G. duodenalis* assemblages A-F from worldwide and the *G. microti* and *G. muris* especies.

*Ethics* - This study was approved by the local SISBIO NO 67153-3 (general license for animal collection), and by UFAM CEUA NO 017/2020 (Federal University of Amazonas State, ethics committee for animal use), and by UFAM CEP/CAAE NO 41067414.6.00005020 (Federal University of Amazonas State, research ethics committee).

## RESULTS

In order to determine the assemblages of *G. duodenalis* from individuals and animals living in rural and urban areas of the Amazonas State (Fig. 1), we screened, microscopically, hundreds of humans and dozens of animal samples. From 107 samples (103 humans and four animals) microscopically *Giardia*-positive, 64.5% (69/107) amplified to *BG* and/or *TPI* targets (63/humans and four/animals) and, consequently, were included in the present study (Table).

**TABLE:** Sub-assemblages of *Giardia duodenalis* from human and animals according to *BG* and *TPI* genes

Locality	Id sample (n = 69)	Assemblage	
		*BG* (n = 44)	*TPI* (n = 46)
Autazes	1SF	﹣	AI
Autazes	78SF	﹣	AI
Autazes	93SF	AI	﹣
Autazes	82SF	AI	﹣
Autazes	77SF	AI	﹣
Autazes	75SF	AI	﹣
Autazes	47SF	AI	﹣
Autazes	43SF	AI	﹣
Autazes	22SF	AI	﹣
Boa Vista do Ramos	9B	AI	AII
Boa Vista do Ramos	49B	AI	﹣
Boa Vista do Ramos	14B	AI	﹣
Boa Vista do Ramos	7B	AI	﹣
Boa Vista do Ramos	6B	AI	﹣
Boa Vista do Ramos	33B	AI	﹣
Boa Vista do Ramos	23B	AIII	﹣
Iranduba	258L	﹣	AI
Iranduba	489L	﹣	AI
Iranduba	8L	﹣	AI
Iranduba	9L	﹣	AI
Iranduba	238L	﹣	AII
Iranduba	437L	﹣	AII
Iranduba	18L	﹣	AII
Iranduba	284L	﹣	AII
Iranduba	291L	﹣	AII
Iranduba	287L	﹣	AII
Iranduba	2L	AIII	AII
Iranduba	14L	AIII	﹣
Iranduba	29L	B	BIII
Iranduba	290L	﹣	B
Iranduba	255L	﹣	B
Manaus	69F	B	AI
Manaus	4M	AI	B
Manaus	72F	AI	AI
Manaus	18M	AI	AI
Manaus	76F	AIII	AI
Manaus	22A_CAT*	﹣	AI
Manaus	700S_NHP*	﹣	AI
Manaus	113A_CAT*	﹣	AI
Manaus	11F	﹣	AI
Manaus	63F	﹣	AI
Manaus	16M	AI	﹣
Manaus	70F	AI	﹣
Manaus	67F	AI	﹣
Manaus	66F	AI	﹣
Manaus	49F	AI	﹣
Manaus	64F	AI	﹣
Manaus	8F	AI	﹣
Manaus	13M	AI	AII
Manaus	15M	AII	AII
Manaus	17M	AII	AII
Manaus	23M	AII	AII
Manaus	4F	AII	AII
Manaus	20M	﹣	AII
Manaus	7M	﹣	AII
Manaus	8M	﹣	AII
Manaus	2M	AIII	AII
Manaus	24M	AIII	AII
Manaus	3M	AIII	AII
Manaus	9M	AIII	AII
Manaus	3F	﹣	AII
Manaus	19M	B	BIII
Manaus	22M	BIII	B
Manaus	11M	BIII	B
Manaus	21M	BIII	BIV
Manaus	2F	B	﹣
Manaus	1F	B	﹣
Manaus	10M	﹣	BIII
Manaus	422_NHP*	﹣	BIV

Note: isolates from Iranduba (Lago do Limão community) were obtained in 2018; from Autazes (São Félix community), Boa Vista do Ramos and Manaus were obtained in 2019. *: animal samples; NHP: non-human primate.

Based on *BG* and *TPI* sequences BLAST analyses were conducted and we identified a diversity of sub-assemblages within the *BG* sequences (AI, AII, AIII, and BIII) and *TPI* (AI, AII, BIII, and BIV) (Table). The AI(*BG*) sub-assemblage was the most prevalent (n = 24/36). The second most frequent sub-assemblage was the AIII(*BG*) (n = 8/36). According to the *TPI* genotyping, the AII sub-assemblage was the most prevalent (n = 21/36), followed by AI(n = 15/36).

We characterise homogeneous and heterogeneous genotypes and found the homogeneous genotypes AI/AI(*BG*/*TPI*) and AII/AII(*BG*/*TPI*) and a variety of heterogeneous genotypes occur in urban and rural areas. In addition, we also found heterogeneous genotypes between assemblages A and B (AI/B and B/AI) (Table).

Within *G. duodenalis* from the four animals (two domestic cats and two NHP of the species *A. paniscus* and *Lagothrix cana*), were also identified the assemblages A (*TPI*) and B (*TPI*). The highest abundance was AI (one NHP, *A. paniscus*, and two cats). The other sub-assemblage BIV was found in one NHP (*L. cana*) (Table). The identity values between assemblage A animal sequences: 113A Cat AI, 22A Cat AI, 700S Monkey AI, relative to the closest human sequences (KF843947, L02120, KP687796 and 489 AI) were 99.55%, 99, 78%, 99.10% respectively. The monkey sequence identified as assemblage B (422M Monkey B4) showed an identity of 99.78% in relation to the closest human sequence (MG754394).

**Fig. 2:**
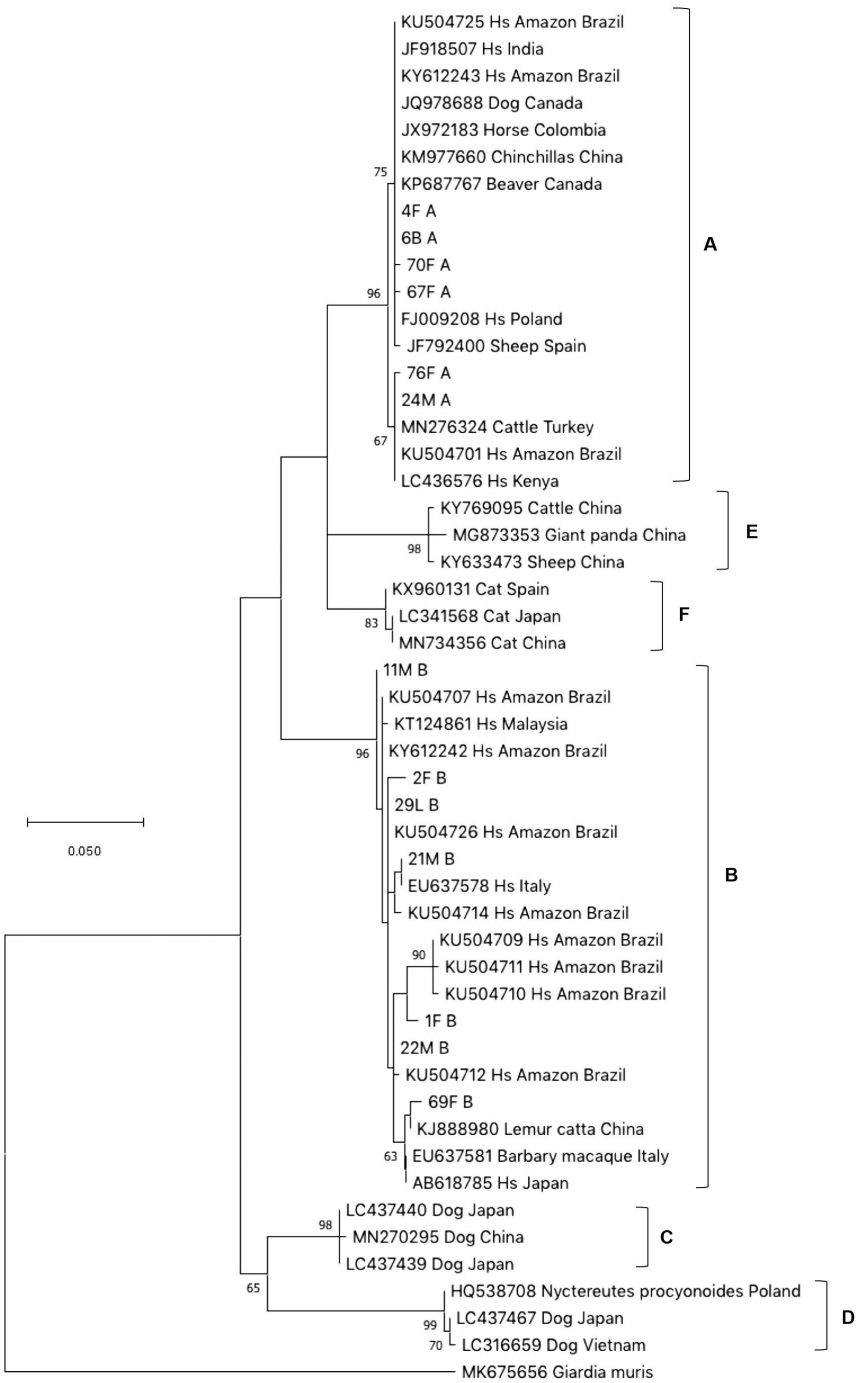
maximum-likelihood phylogenetic tree based on *BG* (445 bp). 1,000 Bootstrap values. These sequences are submitted to GenBank (accession numbers: (*BG*) MZ822137-76). Hs: *Homo sapiens*.

**Fig. 3:**
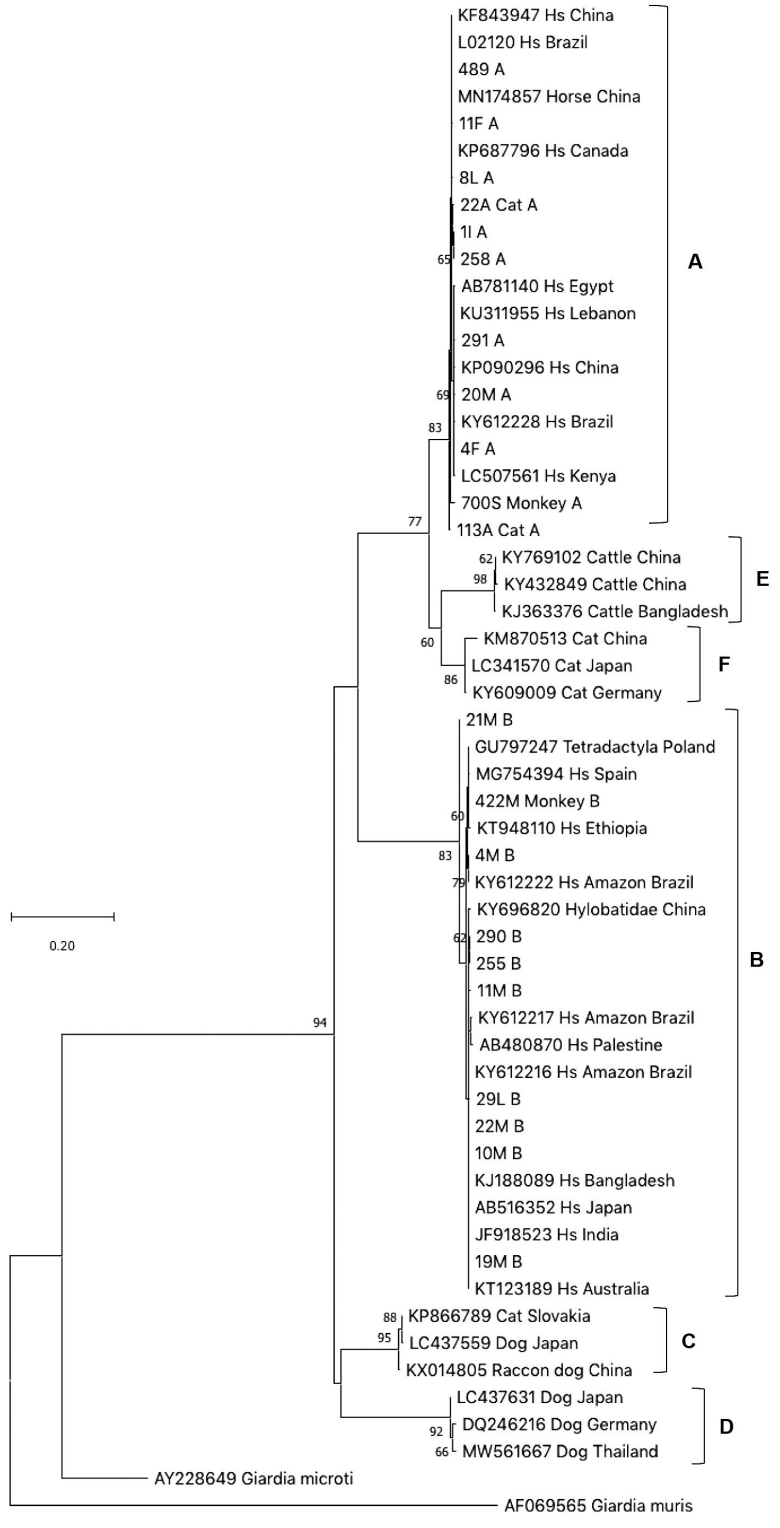
maximum-likelihood phylogenetic tree based on *TPI* (428 bp). 1,000 Bootstrap values. These sequences are submitted to GenBank (accession numbers: (*TPI*) MZ822177-222). Hs: *Homo sapiens*.

We performed a phylogenetic analysis for *BG* and *TPI*, and the *BG* and *TPI* sequences from this study clustered with worldwide sequences belonging to assemblage A or B (Figs 2-3). About 80% of the genotypes belonged to assemblage A: (36/44) and (36/46) for *BG* and *TPI*, respectively, in both urban and rural areas (Table).

## DISCUSSION

This study revealed *G. duodenalis* assemblages A and B in human stools and wild and companion animals, living in Amazonas State, Brazil, which belong to the Amazon biome. In Brazil, the few studies characterising *Giardia* assemblages suggest regional differences in the prevalence of circulating assemblages.^8^ ^,^ ^16^ Considering only larger population studies, assemblage A was most frequently identified in Southeastern Brazil,^6^ ^,^ ^17^ ^,^ ^18^ but assemblage B has been identified as prevalent in other Brazilian regions.^10^ ^,^ ^19^ Interestingly, in the only genotyping study of *G. duodenalis* in Amazonas/Brazil, conducted in Santa Izabel do Rio Negro, an area inhabited mainly by indigenous peoples, assemblage B was predominant.^9^ A similar scenario occurred among indigenous peoples in Mato Grosso, Midwest Brazil, where assemblage B was also predominant.^10^ Globally, most studies showed a higher prevalence of assemblage B^20^ with the exception of Australia, where A was predominant.^4^

The diversity of sub-assemblages identify here, was also been demonstrate in other studies.^10^ ^,^ ^21^ ^,^ ^22^ ^,^ ^23^ Likewise, the highest frequency of the AI (*BG*) sub-assemblage has also been identified in humans in Southeastern Brazil,^17^ ^,^ ^18^ however, worldwide, the sub-assemblage AI(*BG*) is rare in humans.^24^ ^,^ ^25^ ^,^ ^26^ The second sub-assemblage most frequent in this study AIII(*BG*) is also the most prevalent in the indigenous population of Brazilian Amazon.^9^ ^,^ ^10^ In other Brazilian regions, AIII(*BG*) is a rare genotype, identified only in Paraná/ South Brazil (n = 1/10, *BG*).^27^ Globally, this genotype was identified in Mozambique/Africa (n = 8/14, *BG*),^22^ Malaysia (n = 6/18, *BG*)^28^ and France (n = 6/14, *BG*).^25^ According to the *TPI* genotyping, the AII sub-assemblage was the most prevalent, followed by AI. This scenario, in which AII(*TPI*) predominates, has also been shown in other studies in the Southeast Brazilian region,^21^ ^,^ ^29^ and worldwide in Colombia,^23^ Czech Republic,^24^ France^25^ and Malaysia.^28^

Having recovered both genes (*BG* and *TPI*) from a number of samples, we were able to characterise homogeneous and heterogeneous genotypes. The homogeneous genotypes AI/AI(*BG*/*TPI*) and AII/AII(*BG*/*TPI*) and a variety of heterogeneous genotypes occur in urban and rural areas. Interestingly, all B assemblages were heterogeneous and, in fact, it has been suggested that the B assemblage is a heterogeneous genotype.^30^ In addition, we also found heterogeneous genotypes between assemblages A and B (AI/B and B/AI). To extend this scenario, we reanalysed the data of Nunes et al.^9^ to identify homogeneous and heterogeneous genotypes in indigenous peoples from the Amazonas. Interestingly, based on the sequences from their study, the presence of three homogeneous B(*BG*/*TPI*) genotypes was detected, at least when these two genes were considered. The most likely hypothesis for the identification of heterogeneous *G. duodenalis* assemblages is the occurrence of mixed infections, especially in studies of endemic countries.^20^ However, recombination events between assemblages should also be considered, as there are a number of clues suggesting *Giardia* sexual reproduction.^31^

The same assemblages A and B also identified in four animals (two domestic cats and two NHP of the species *A. paniscus* and *L. cana*), according *TPI*. Interestingly, the highest abundance was AI (one NHP, *A. paniscus*, and two cats), the second most abundant sub-assemblage among humans in this study. The other sub-assemblage BIV, found in one NHP (*L. cana*), also was identified in one human/Manaus. This piece of work represents the first data on *Giardia* assemblages and sub-assemblages in animals in the Brazilian Amazon biome,^8^ region extraordinarily rich in biodiversity, having the greatest mammals diversity in Brazil, with many of these animals being unique to the Amazon (endemic). Unexpectedly, the sub-assemblages (*TPI*) identified in the wild animals from Amazon biome were also found in animals (dog/cat) in the Southeast region^32^ ^,^ ^33^ and in humans.^21^ Worldwide, sub-assemblages AI(*TPI*) have been found in cats/EUA^34^ and in dogs, cats, and horses/China.^35^ ^,^ ^36^ Our results indicate that, in Amazonas, there is a same assemblage circulating between animals and humans. However, this evidence doesn’t allow to infer on the transmission of this infection between these humans and animals. Assemblages A and B are considered zoonotic,^31^ and here, in the Amazonas/Brazil, we also show that humans and animals share *G. duodenalis* genotypes found in cosmopolitan regions around the world.
